# Oncological Outcomes of Non-clear Cell Renal Cell Carcinomas: A Retrospective Study From a Tertiary Care Center

**DOI:** 10.7759/cureus.89022

**Published:** 2025-07-29

**Authors:** Vijay K Bhargava, Shaunak Valame, Harsh Sahu, Vikas Gupta, Rachna Jain, Shweta Azad, Dhruti Manek, Vandna Bharati

**Affiliations:** 1 Medical Oncology, Jawaharlal Nehru Cancer Hospital and Research Centre, Bhopal, IND; 2 Community Medicine, Government Medical College, Seoni, Seoni, IND; 3 Pathology, Jawaharlal Nehru Cancer Hospital and Research Centre, Bhopal, IND

**Keywords:** chromophobe rcc, non-clear cell renal carcinoma, oncological outcomes, papillary rcc, survival

## Abstract

Background

Non-clear cell renal cell carcinomas (nccRCCs) are a histologically heterogeneous and clinically underrepresented group of renal malignancies, distinct from clear cell RCC in morphology, molecular profile, and prognosis. Despite accounting for 20-30% of RCCs, nccRCC subtypes are infrequently studied, especially in the Indian population. This study aimed to evaluate the clinicopathological features, immunohistochemical patterns, and oncological outcomes of primary nccRCCs in a tertiary care setting.

Methods

This was a retrospective, single-center study including 37 adult patients diagnosed with histologically confirmed nccRCC between January 2015 and December 2024. Subtypes analyzed included papillary RCC, chromophobe RCC, collecting duct carcinoma, Xp11.2 translocation RCC, mucinous tubular and spindle cell carcinoma (MTSCC), unclassified RCC, and other rare variants. Demographic, clinical, pathological, and immunohistochemical (IHC) data were evaluated. Kaplan-Meier analysis was used to estimate disease-free survival (DFS) and overall survival (OS), with comparisons between subtypes made using the log-rank test and Chi-square/Fisher’s exact test.

Results

The cohort had a mean age of 54.3 ± 11.2 years and was predominantly male (67.6%). Papillary RCC (37.8%) and chromophobe RCC (21.6%) were the most common subtypes. Collecting duct carcinoma exhibited significantly higher rates of distant metastasis (80%, p = 0.014) and mortality (80%, p = 0.005), confirming its aggressive nature, as observed in prior studies. Median DFS and OS were 19 months (IQR: 12-36) and 31 months (IQR: 18-48), respectively. Five-year survival was highest for chromophobe RCC (87.5%) and lowest for collecting duct carcinoma (0%, p = 0.001). CK7 and CD117 were highly expressed in papillary and chromophobe RCCs, respectively, aiding subtype identification. The overall mortality rate was 37.8%. The small sample size and single-institution scope were key limitations.

Conclusion

This study demonstrates the prognostic variability among nccRCC subtypes, with papillary and chromophobe RCC showing relatively favorable outcomes, and collecting duct carcinoma associated with poor survival. Histological subtype remains the cornerstone of prognosis and therapeutic decision-making. Subtype-specific management strategies, early recognition through IHC, and incorporation of molecular profiling are essential to optimize outcomes. Larger, multicentric prospective studies are warranted to validate these findings and improve clinical guidance in resource-limited settings.

## Introduction

Renal cell carcinoma (RCC) accounts for approximately 2-3% of all adult malignancies worldwide and is the most common form of kidney cancer, with global incidence steadily increasing due to the widespread use of advanced imaging and incidental detection [1,2]. As per GLOBOCAN 2020 estimates, over 431,000 new RCC cases and 179,000 deaths were reported globally [3]. In India, RCC represents around 2.4% of all male cancers and 1.2% of female cancers, with a rising trend linked to improved diagnostic access [4].

RCC is broadly classified into two major categories based on histopathology: clear cell RCC (ccRCC), which constitutes 70-80% of cases, and non-clear cell RCCs (nccRCCs), comprising approximately 20-30% [5,6]. While ccRCC is well-characterized, the nccRCC group includes histologically and molecularly diverse subtypes such as papillary RCC (10-15%), chromophobe RCC (4-6%), collecting duct carcinoma (<1%), renal medullary carcinoma (<0.5%), translocation-associated RCC (1-4%), mucinous tubular and spindle cell carcinoma, and unclassified RCC (1-5%) [7]. These entities differ significantly in their morphological features, genetic drivers, natural history, and treatment responsiveness. For instance, type 1 papillary RCC typically follows a more indolent course, whereas type 2 papillary RCC and collecting duct carcinoma are known for their aggressive clinical behavior and poor outcomes [8]. Chromophobe RCC, although rare, generally exhibits low metastatic potential and favorable survival [9].

Despite this biological heterogeneity, treatment algorithms for nccRCCs are frequently extrapolated from ccRCC-centric clinical trials due to the underrepresentation of nccRCC patients in randomized controlled studies [10]. Targeted therapies such as tyrosine kinase inhibitors (TKIs), mTOR inhibitors, and, more recently, immune checkpoint inhibitors have shown inconsistent efficacy across nccRCC subtypes, emphasizing the need for subtype-specific therapeutic strategies [10]. Furthermore, prognosis varies widely depending on histological subtype, tumor grade, and molecular characteristics.

There is limited high-quality data on nccRCCs, particularly in the Indian population. Most existing literature is based on small institutional series with wide heterogeneity in subtype prevalence, follow-up duration, and survival outcomes [11-13]. For example, Roy S et al. reported on patients with nccRCC and emphasized the predominance of papillary and chromophobe subtypes but lacked long-term follow-up [11]. Similarly, Naik P et al. studied nccRCCs in a central Indian cohort and highlighted histological diversity and outcome variability, but were limited by small sample size and retrospective design [13]. These studies underscore the persistent knowledge gap and the need for robust data from Indian centers.

Additionally, population differences in genetics, environmental exposures, healthcare access, and treatment availability further justify the need for regional studies. Addressing these disparities is crucial for refining prognostic models and guiding contextually appropriate clinical decisions.

Therefore, this study was undertaken with the primary objective of evaluating the oncological outcomes, specifically disease-free survival (DFS) and overall survival (OS), of patients diagnosed with nccRCCs at a tertiary care center in Central India.

The secondary objectives of this study were to analyze the demographic, clinical, pathological, and immunohistochemical profiles of patients with nccRCC; to assess recurrence patterns and treatment modalities employed across various histological subtypes; and to compare subtype-specific survival outcomes while identifying key prognostic indicators. By focusing on a well-defined Indian cohort with detailed histopathological review and survival follow-up, this study aims to contribute meaningful insights to the limited evidence base on nccRCCs in resource-limited settings.

## Materials and methods

Study design and setting

This retrospective observational study was conducted in the Department of Oncology for a period of 5 months (between January 2025 to May 2025) at a tertiary care academic hospital in Central India. The study aimed to evaluate oncological outcomes in patients diagnosed with primary renal malignancies, excluding ccRCC. The Institutional Ethics Committee approved the study protocol (Approval Number: IEC/2025/009). Given the retrospective design and use of anonymized clinical data, a waiver of informed consent was granted by the Ethics Committee in accordance with institutional policy and the ethical standards of the Declaration of Helsinki and its amendments.

Study population

All adult patients (≥18 years) who underwent partial or radical nephrectomy between January 2015 and December 2024 and were histologically diagnosed with nccRCC were included. The classification of renal tumors was based on the 2022 WHO classification [14], encompassing papillary RCC (types 1 and 2), chromophobe RCC, collecting duct carcinoma, renal medullary carcinoma, MiT family translocation RCC, and unclassified RCC.

Patients were excluded if they had: clear cell RCC as the predominant histology; secondary renal involvement from non-renal primary malignancies; incomplete clinical, pathological, or follow-up records; and loss to follow-up within three months post-surgery.

The rationale for the three-month minimum follow-up threshold was to ensure adequate postoperative surveillance for meaningful survival analysis and to exclude early attrition unrelated to disease biology. This decision was made to minimize misclassification bias in survival endpoints.

Data collection

Data were extracted from institutional electronic medical records, operative logs, and pathology databases (between January 2025 to May 2025) using a structured proforma. The variables collected included demographics (age, sex, comorbidities such as hypertension and diabetes mellitus, and smoking history); clinical characteristics (presenting symptoms, tumor laterality, and imaging features); surgical details (type of nephrectomy, partial or radical, surgical approach including open, laparoscopic, or robotic, lymphadenectomy status, operative time, estimated blood loss, and surgical margins); and histopathology (tumor subtype, size, Fuhrman grade where applicable, presence of necrosis, sarcomatoid or rhabdoid differentiation, lymphovascular invasion, and pathological TNM staging according to the AJCC 8th edition) [15].

Data were independently extracted and cross-verified by two investigators to ensure accuracy. A randomly selected 15% subset of cases was double-checked for validation. In instances where key clinical, pathological, or follow-up variables were missing, those entries were excluded from the relevant analyses. Overall, missing data were minimal (<5%), and no imputation techniques were employed due to the low proportion of missing values.

Treatment and follow-up protocol

Patients were managed according to institutional protocols, which were consistent with internationally accepted standards and tailored based on histological subtype, tumor stage, and patient performance status. Adjuvant therapy was considered in cases with high-grade tumors (Fuhrman grade G3-G4), sarcomatoid or rhabdoid differentiation, pathologic T3/T4 disease, and nodal or distant metastasis at presentation or during follow-up.

Adjuvant therapy decisions were guided by histological subtype, tumor grade, and pathological stage. Indications included Fuhrman grade 3 or 4, lymphovascular invasion, positive surgical margins, nodal involvement, or T3/T4 disease. Initially, from 2015 to 2018, systemic therapy primarily included chemotherapy (e.g., gemcitabine-platinum combinations) for aggressive subtypes such as collecting duct carcinoma. From 2019 onward, targeted therapies such as tyrosine kinase inhibitors (sunitinib and pazopanib) and mTOR inhibitors (everolimus) were incorporated. Immune checkpoint inhibitors became selectively available and were used in high-risk patients from 2021 onward, reflecting evolving national oncology protocols and access to newer agents. Patients were followed every 3 months for the first 2 years, biannually until 5 years, and annually thereafter. Follow-up evaluations included clinical examination, renal function tests, abdominal imaging (ultrasound, contrast-enhanced CT or MRI), and chest imaging (X-ray or CT thorax). Recurrence was defined as radiological or histological evidence of disease at the surgical bed, lymph nodes, or distant sites.

Outcome measures

The primary outcomes were DFS, defined as the time from nephrectomy to the first documented recurrence or last disease-free follow-up, and OS, defined as the time from nephrectomy to death from any cause or last follow-up. Secondary outcomes included recurrence rates by histological subtype and TNM stage, and patterns of local and distant relapse.

Statistical analysis

All analyses were conducted using IBM SPSS Statistics for Windows, Version 20.0 (IBM Corp., Armonk, NY, USA). Continuous variables were expressed as mean ± SD, while categorical variables were summarized as frequencies and percentages. Comparative analyses between subgroups were performed using the Chi-square test or Fisher’s exact test for categorical variables. Kaplan-Meier survival analysis was used to estimate DFS and OS. Median survival times were reported with interquartile ranges. Differences between survival curves of histological subtypes were assessed using the log-rank test. Due to the limited sample size and low number of outcome events in certain subgroups, multivariate Cox regression was not performed, as it would risk model overfitting and unreliable hazard estimates. A two-tailed p-value < 0.05 was considered statistically significant.

## Results

A total of 37 patients with histologically confirmed nccRCC were included in the study. The mean age of the cohort was 54.3 ± 11.2 years, with a predominance of patients aged between 40 and 60 years. Males constituted 67.6% of the cohort, reflecting a male-to-female ratio of approximately 2:1. Tumors were slightly more common on the right side. The majority of patients (78.4%) presented with symptoms, most commonly flank pain. Incidental detection was observed in 21.6% of cases. Hypertension and diabetes mellitus were the most frequent comorbidities. A history of smoking was reported in over one-fourth of the cohort (Table 1).

**Table 1:** Demographic and clinical profile of patients with primary non-clear cell renal cell carcinomas (ncRCCs).

Variable	Frequency (%) / Mean ± SD
Age (years)	54.3 ± 11.2
Age groups	
< 40 years	4 (10.8%)
40–60 years	20 (54.1%)
> 60 years	13 (35.1%)
Gender	
Male	25 (67.6%)
Female	12 (32.4%)
Side of tumor	
Right	20 (54.1%)
Left	17 (45.9%)
Symptomatic presentation	29 (78.4%)
Most common symptom (flank pain)	19 (51.4%)
Incidental detection	8 (21.6%)
Comorbidities	
Hypertension	18 (48.6%)
Diabetes mellitus	12 (32.4%)
Chronic kidney disease (CKD)	4 (10.8%)
Smoking history	10 (27.0%)

**Figure 1:**
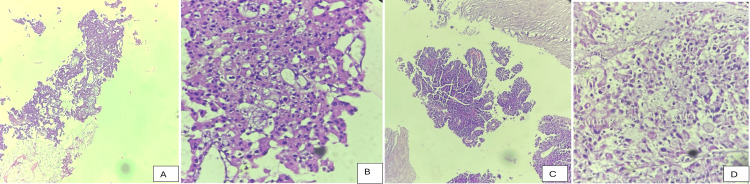
A, B, C: Papillary RCC (10×, 40×, 10× magnification, respectively): Images depict various patterns of papillary RCC comprising tubules, papillae, and sheets. Focal areas show a foamy appearance. In image C, fibrovascular cores are identified. The tumor cells have abundant eosinophilic cytoplasm. WHO/ISUP Grade 2: D: Chromophobe renal cell carcinoma (40× magnification): the image depicts tumor cells arranged in solid sheets, separated by thickened vascular septa. A nested pattern is also identified. The cytoplasm appears translucent and finely reticulated. Some larger cells show voluminous eosinophilic, granular to foamy cytoplasm. Hyperchromatic nuclei and focal perinuclear cytoplasmic haloes are also noted. ISUP: International Society of Urological Pathology; RCC: Renal Cell Carcinoma.

Histopathological evaluation identified papillary RCC (pRCC) as the most frequently encountered subtype, comprising 37.8% of cases, followed by chromophobe RCC (chRCC) (21.6%) and collecting duct carcinoma (CDC) (13.5%). Less frequent subtypes included Xp11.2 translocation RCC, mucinous tubular and spindle cell carcinoma (MTSCC), unclassified RCC, and other rare variants, each contributing to the overall heterogeneity of the cohort. The mean tumor diameter was 6.8 ± 2.5 cm. Tumors measuring 4-7 cm were most common (45.9%), while 37.8% were larger than 7 cm and 16.2% measured less than 4 cm. Approximately 51.4% of tumors were graded Fuhrman G3-G4, indicating a high proportion of aggressive histology. Similarly, advanced pathological stage (T3-T4) was observed in 48.6% of patients. Adverse histological features such as lymphovascular invasion (27%), sarcomatoid differentiation (13.5%), and tumor necrosis (32.4%) were noted across subtypes (Table 2 and Figure 1).

**Table 2:** Histopathological characteristics of tumors in patients with non–clear cell renal cell carcinoma (nccRCC). Rare variants included tubulocystic RCC, medullary carcinoma, and MiT family translocation RCC. RCC: Renal cell carcinoma; G: Grade (Fuhrman nuclear grade); T: Tumor stage (AJCC 8th edition).

Variable	Frequency (%) / Mean ± SD
Subtypes	
Papillary RCC	14 (37.8%)
Chromophobe RCC	8 (21.6%)
Collecting duct carcinoma	5 (13.5%)
Xp11.2 translocation RCC	2 (5.4%)
Mucinous tubular and spindle cell	2 (5.4%)
Unclassified RCC	3 (8.1%)
Other rare variants	3 (8.1%)
Tumor size (cm)	6.8 ± 2.5
Tumor size category	
< 4 cm	6 (16.2%)
4–7 cm	17 (45.9%)
> 7 cm	14 (37.8%)
Histological grade (Fuhrman)	
G1–2	18 (48.6%)
G3–4	19 (51.4%)
Pathological T stage	
T1	14 (37.8%)
T2	5 (13.5%)
T3–T4	18 (48.6%)
Lymphovascular invasion	10 (27.0%)
Sarcomatoid differentiation	5 (13.5%)
Tumor necrosis	12 (32.4%)

Immunohistochemical analysis revealed subtype-specific expression patterns that support both diagnostic and prognostic stratification. CK7 was commonly expressed across tumors, particularly in chRCC and pRCC, reflecting its utility in identifying epithelial lineage, although the intergroup difference was not statistically significant. CD117 demonstrated a strong and statistically significant association with chRCC (p < 0.001), reinforcing its role as a key marker for this subtype. Similarly, alpha-methylacyl-CoA racemase (AMACR) was significantly overexpressed in pRCC (p = 0.003), aligning with its established diagnostic relevance for papillary histology. Vimentin expression, while highest in CDC, lacked statistical significance, indicating limited discriminatory utility. Uniform retention of INI-1 expression across all subtypes effectively ruled out renal medullary carcinoma in the cohort (Table 3).

**Table 3:** Immunohistochemical profile of various nccRCC subtypes. IHC was performed in 31 out of 37 patients. RCC: Renal cell carcinoma; CK7: Cytokeratin 7; CD117: Cluster of differentiation 117; AMACR: Alpha-methylacyl-CoA racemase; INI-1: Integrase interactor 1. INI-1 was retained in all cases; hence, p-value not applicable. A p-value < 0.05 was considered statistically significant.

Marker	Papillary RCC (n = 14)	Chromophobe RCC (n = 8)	Collecting Duct (n = 5)	Others (n = 4)	p-value	Test of significance
Frequency (%)						
CK7 positive	11 (78.6%)	7 (87.5%)	3 (60.0%)	3 (75.0%)	0.51	χ² = 1.71
CD117 positive	2 (14.3%)	7 (87.5%)	1 (20.0%)	1 (25.0%)	< 0.001	χ² = 17.65
AMACR positive	10 (71.4%)	1 (12.5%)	1 (20.0%)	2 (50.0%)	0.003	χ² = 11.63
Vimentin	9 (64.3%)	3 (37.5%)	4 (80.0%)	2 (50.0%)	0.26	χ² = 3.25
INI-1 retained	14 (100.0%)	8 (100.0%)	5 (100.0%)	4 (100.0%)	-	-

Among the 37 patients, radical nephrectomy was performed in 75.7%, and partial nephrectomy in 24.3%. The surgical approach was open in 56.8%, laparoscopic in 32.4%, and robotic in 10.8% of cases. Lymph node dissection was performed in 59.5%. The mean operative time was 172.4 ± 36.8 minutes, with mean blood loss of 324.5 ± 121.2 mL. The median postoperative stay was 6 days, with no 30-day mortality. Postoperative complications occurred in 32.4% of patients, mostly low-grade. Recurrence rates were highest in CDC (80%) and lowest in chRCC (12.5%). Adjuvant therapy was administered to 32.4% of patients, while 67.6% received no adjuvant treatment (Table 4).

**Table 4:** Surgical and postoperative parameters in patients undergoing nephrectomy for nccRCC. Sunitinib = 1, Nivolumab = 1. RCC: Renal cell carcinoma; TKIs: Tyrosine kinase inhibitors; Clavien: Clavien–Dindo classification.

Variable	Frequency (%) / Mean ± SD
Surgical procedure (nephrectomy)	
Radical	28 (75.7%)
Partial	9 (24.3%)
Surgical approach	
Open	21 (56.8%)
Laparoscopic	12 (32.4%)
Robotic	4 (10.8%)
Lymph node dissection	22 (59.5%)
Operative time (minutes)	172.4 ± 36.8
Mean blood loss (mL)	324.5 ± 121.2
Median postoperative stay (days)	6 (IQR: 5–9)
30-day postoperative mortality	0 (0.0%)
Postoperative complications	12 (32.4%)
Surgical site infection	3 (8.1%)
Bleeding requiring transfusion	4 (10.8%)
Urinary leak	1 (2.7%)
Fever (Grade I)	6 (16.2%)
Pneumonia	2 (5.4%)
Clavien grade (postoperative complications)	
Grade I	6 (16.2%)
Grade II	4 (10.8%)
Grade IIIa	2 (5.4%)
Grade IIIb	3 (8.1%)
Grade IV or V	0 (0.0%)
Subtype recurrence	
Papillary RCC	3 (21.4%); Lung (2), Bone (1)
Chromophobe RCC	1 (12.5%); Lung
Collecting duct	4 (80.0%); Lung, Liver, Bone
Others	5 (50.0%); Lung, Liver
Adjuvant therapy	
TKIs	3 (8.1%)
Chemotherapy (gemcitabine + cisplatin)	4 (10.8%)
Immune checkpoint inhibitor	2 (5.4%)
Radiation therapy (for metastasis)	3 (8.1%)
None	25 (67.6%)

**Figure 2:**
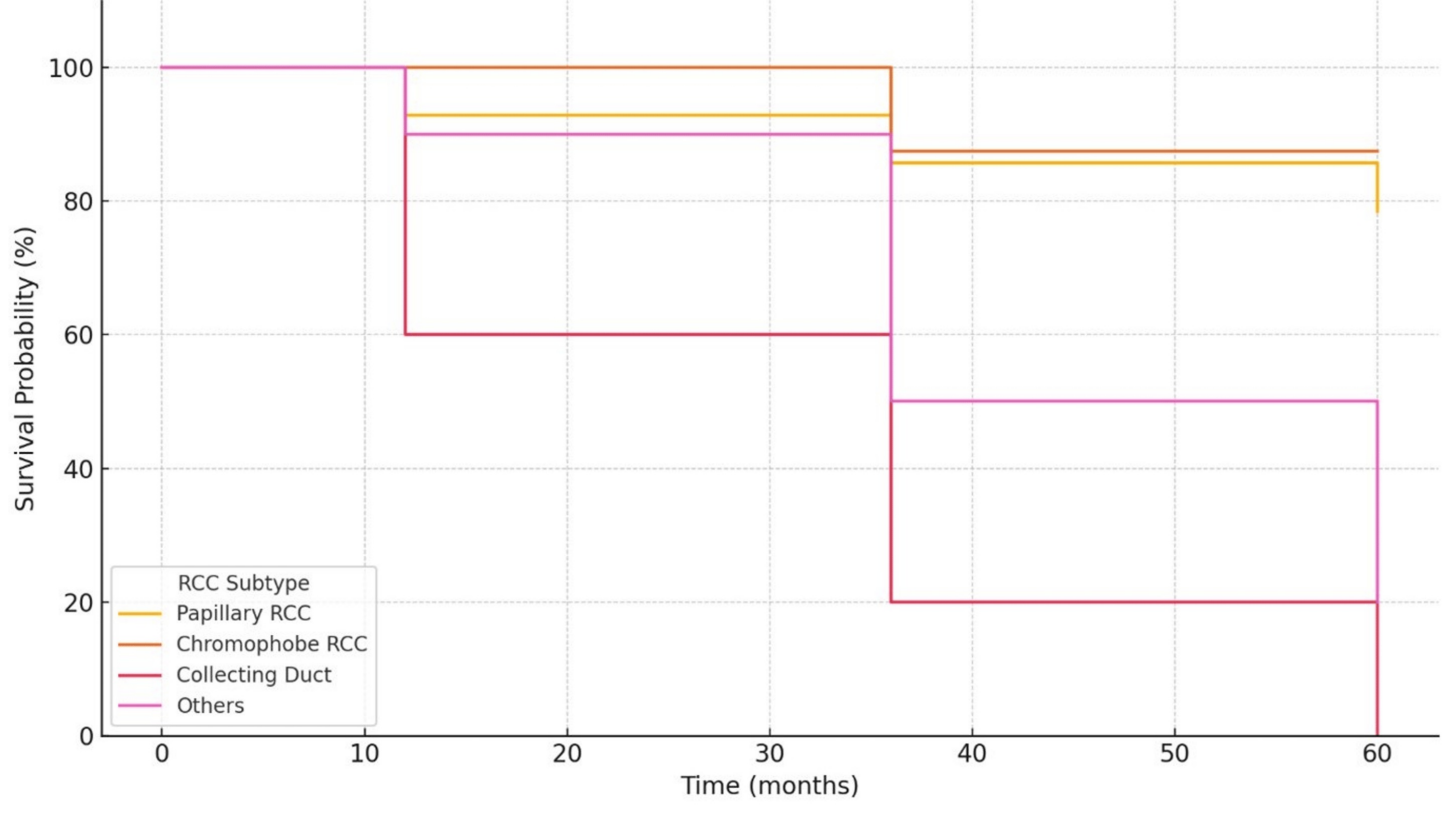
Kaplan-Meier plot showing survival probabilities over time (in months) for different non-clear cell renal cell carcinoma subtypes.

Oncological outcomes demonstrated substantial variability across histological subtypes. Overall, local recurrence was observed in 18.9% of patients, with the highest rate seen in CDC (40.0%). However, this difference was not statistically significant (p = 0.318). Distant metastases occurred in 32.4% of cases, significantly more frequent in CDC (80.0%) and other rare variants (40.0%) compared to pRCC and chRCC (p = 0.014). The overall mortality rate was 37.8%, with a disproportionate burden observed in CDC (80.0%) and rare subtypes (60.0%), which was statistically significant (p = 0.005). In terms of survival outcomes, median DFS and OS were notably shorter in aggressive subtypes. Median DFS was 6 months (IQR: 5-9) for CDC, 27 months (IQR: 16-39) for pRCC, and not reached for chRCC at the last follow-up. Similarly, OS was 12 months (IQR: 10-19) in CDC, 33 months (IQR: 21-46) in pRCC, and not reached in chRCC (Table 5). Five-year survival rates further reflected this disparity, 0% in CDC, 20% in other rare variants, compared to 78.5% in pRCC and 87.5% in chRCC, with statistically significant differences (p = 0.001) (Table 5 and Figure 2).

**Table 5:** Oncological outcomes and survival analysis by histological subtype of nccRCC. RCC: Renal cell carcinoma; DFS: Disease-free survival; OS: Overall survival; IQR: Interquartile range; nccRCC: Non-clear cell renal cell carcinoma. Statistical comparisons for categorical variables were performed using the Chi-square test; Kaplan-Meier survival analysis with the log-rank test was used for survival metrics. A p-value <0.05 was considered statistically significant.

Outcome	Total (n = 37)	Papillary RCC (n = 14)	Chromophobe RCC (n = 8)	Collecting Duct (n = 5)	Others (n = 10)	p-value	Test of significance
Local recurrence, n (%)	7 (18.9%)	2 (14.3%)	1 (12.5%)	2 (40.0%)	2 (20.0%)	0.318	χ² = 3.74
Distant metastasis, n (%)	12 (32.4%)	3 (21.4%)	1 (12.5%)	4 (80.0%)	4 (40.0%)	0.014	χ² = 10.56
Mortality, n (%)	14 (37.8%)	3 (21.4%)	1 (12.5%)	4 (80.0%)	6 (60.0%)	0.005	χ² = 12.72
DFS (months), median (IQR)	19 (6-57)	27 (11-59)	Not reached	6 (3-10)	10 (4-17)	0.002	Log-rank χ² = 13.49
OS (months), median (IQR)	31 (11-65)	33 (19-64)	Not reached	12 (7-22)	25 (9-36)	0.008	Log-rank χ² = 11.67
1-year survival (%)	89.20%	92.90%	100.00%	60.00%	90.00%	0.038	Log-rank χ² = 8.46
3-year survival (%)	65.30%	85.70%	87.50%	20.00%	50.00%	0.011	Log-rank χ² = 10.91
5-year survival (%)	43.20%	78.50%	87.50%	0.00%	20.00%	0.001	Log-rank χ² = 15.98

## Discussion

Primary renal malignancies excluding ccRCC, commonly grouped as nccRCCs, comprise a histologically diverse and biologically heterogeneous group of tumors that differ significantly in prognosis, molecular profile, and treatment responsiveness. The mean age of presentation in our cohort was 54.3 ± 11.2 years, with a predominant age range of 40-60 years (54.1%) and a male preponderance (67.6%). This age and gender distribution closely mirrors findings from the review by Naik P et al., where the mean age for nccRCC was 56 years with a male-to-female ratio of approximately 2:1 [13]. Most patients (78.4%) were symptomatic at presentation, with flank pain being the most common symptom (51.4%). These findings are in line with global data, where symptomatic presentation remains more frequent among nccRCC subtypes due to their tendency for larger size and aggressive behavior at diagnosis [16,17]. In the study by Nouiakh L et al. among patients with nccRCC, the most common symptom was low back pain (reported by all patients), followed by hematuria in one-third of cases [18].

Notably, 48.6% of patients had comorbid hypertension and 32.4% had diabetes mellitus, common metabolic conditions that have been implicated as risk factors for RCC development through pathways involving chronic inflammation and insulin-like growth factor signaling [19]. A history of smoking was noted in 27% of cases, reinforcing its established role as a modifiable risk factor for RCC [20].

Among the nccRCC subtypes in our study, pRCC was the most frequent (37.8%), followed by cchRCC (21.6%), CDC (13.5%), Xp11.2 translocation RCC (5.4%), MTSCC (5.4%), and others (8.1%). This distribution aligns with prior literature by Angori S et al., where pRCC and chRCC constitute the bulk of nccRCC cases [21]. Roy S et al. have reported slightly lower incidences of chromophobe RCC, constituting 15% of nccRCCs [11].

Tumor size was variable, with a mean diameter of 6.8 ± 2.5 cm; 45.9% of tumors measured between 4-7 cm, and 37.8% exceeded 7 cm. High-grade histology (Fuhrman grade G3-G4) was present in 51.4% of cases, and advanced pathological stage (T3-T4) was noted in 48.6%, findings that suggest a significant proportion of nccRCCs present with aggressive features. This has been echoed in reviews by Barthélémy P et al. and Msaouel P et al., who observed that nccRCCs often present at higher stages than ccRCCs and exhibit distinct biological behaviors [22,23].

Sarcomatoid differentiation (13.5%), lymphovascular invasion (27%), and tumor necrosis (32.4%) were frequent, especially among collecting duct and unclassified variants, features previously shown to correlate with poor prognosis [24]. Tumor necrosis has been reported as a key adverse factor in pRCC and CDC, as shown by Lobo J et al. [25].

Immunohistochemical profiling revealed subtype-specific marker expression. CK7 was highly expressed in pRCC (78.6%) and chRCC (87.5%), consistent with established diagnostic algorithms [26]. CD117 was markedly elevated in chRCC (87.5%, p < 0.001), a pattern well documented in multiple studies including Amin J et al., who described CD117 as a distinguishing marker for chromophobe histology [27]. Conversely, AMACR showed strong expression in pRCC (71.4%, p = 0.003), reinforcing its utility in differentiating pRCC from other subtypes [28]. INI-1 expression was retained in all cases, excluding renal medullary carcinoma from the differential diagnosis. These patterns not only assist diagnosis but may also have future implications for targeted therapy selection.

The surgical management in our cohort predominantly involved radical nephrectomy (75.7%), which aligns with the aggressive nature and advanced presentation of nccRCCs, particularly papillary and collecting duct variants. The choice of open surgical approach in over half the cases (56.8%) reflects both tumor complexity and resource settings typical of tertiary Indian centers. The absence of 30-day postoperative mortality and acceptable complication rates (32.4%) underscore the safety and feasibility of nephrectomy in this group. Most complications were Clavien grade I-II, consistent with Xue Z et al., who reported low perioperative morbidity for nephrectomy in nccRCC patients [29]. Despite the high-risk nature of these tumors, adjuvant therapy was administered in only 32.4% of cases, with 8.1% receiving tyrosine kinase inhibitors, and 10.8% receiving chemotherapy (gemcitabine and cisplatin), possibly due to limited efficacy data and resource constraints, a trend also seen in the Indian study by Roy S et al. [11].

Oncological outcomes demonstrated substantial heterogeneity across subtypes. Overall, local recurrence was seen in 18.9% of patients, and distant metastasis occurred in 32.4%, with significant variation by subtype (p = 0.014). Collecting duct carcinoma had the highest metastatic rate (80%) and local recurrence (40%), reaffirming its reputation as the most aggressive subtype of nccRCC. These figures are consistent with the study by Xue Z et al., where 5-year survival in CDC was below 10% [29].

The overall mortality rate in our study was 37.8%, again with marked subtype variability, highest in CDC (80%) and “other variants” (60%), compared to papillary (21.4%) and chromophobe RCC (12.5%) (p = 0.005). Median DFS was 19 months (range: 6-57), but subtype-specific differences were significant: 27 months for pRCC, not reached for chRCC, and only 6 months for CDC (p = 0.002). Median OS mirrored this trend, 31 months overall, but only 12 months in CDC compared to 33 months in pRCC and not reached in chRCC (p = 0.008). These survival figures are in line with those reported by Papanikolaou D et al. and Roldan-Romero JM et al., who highlighted superior prognosis in chromophobe RCC and poor outcomes in CDC and unclassified RCCs [30,31].

Short- and long-term survival data showed steep attrition over time. While the 1-year survival was 89.2%, it dropped to 65.3% at 3 years and 43.2% at 5 years. Notably, 5-year survival for CDC was 0%, and for “other” subtypes only 20%, whereas chromophobe RCC had a 5-year survival of 87.5%, in accordance with studies by Tang C et al. and Qian X et al., showing favorable survival in chRCC (over 80% at 5 years) [32,33].

Interpretation and implications

These findings reinforce the critical role of histological subtype in determining both prognosis and therapeutic direction in nccRCC. Chromophobe RCC, marked by its low nuclear grade, minimal necrosis, and infrequent lymphovascular invasion, consistently demonstrated an indolent clinical course in our cohort, often justifying nephron-sparing approaches in suitable candidates. Conversely, collecting duct carcinoma and unclassified RCCs were associated with high-grade morphology, early metastatic spread, and dismal survival, underscoring the need for early multimodal interventions, including systemic therapy. The integration of immunohistochemical (IHC) profiling, highlighting markers such as CK7, CD117, and AMACR, not only strengthened the diagnostic accuracy of subtypes but also facilitated the correlation between tumor biology and clinical behavior. However, while these markers provide valuable differentiation, it is important to acknowledge that the reproducibility of IHC interpretation can vary based on pathologist experience, antibody specificity, and laboratory standardization. These challenges are especially pronounced in low-resource settings, where access to validated antibodies, quality controls, and subspecialty-trained pathologists may be limited. This underscores the need for capacity-building in oncopathology services and potential incorporation of telepathology or centralized reporting systems to ensure diagnostic consistency.

Limitations

This study, while offering valuable insight into the oncological outcomes of nccRCC subtypes in an Indian tertiary care setting, has several limitations that warrant consideration. First, the retrospective nature of the analysis introduces inherent challenges, including potential inconsistencies in data documentation and the inability to control for all confounding variables, particularly in the context of varied treatment protocols across subtypes. Second, the sample size was relatively small (n = 37), limiting the statistical power to detect differences between rare subtypes such as collecting duct carcinoma, Xp11.2 translocation RCC, and mucinous tubular and spindle cell carcinoma. Third, this was a single-center study and therefore subject to referral bias, as patients managed at tertiary institutions often present with more advanced or atypical disease profiles. Fourth, the exclusion of patients lost to follow-up within three months may have introduced a degree of selection bias, potentially underrepresenting individuals with very aggressive disease or those who are socioeconomically disadvantaged and unable to continue care. This limitation should be considered when interpreting recurrence and short-term survival outcomes.

Furthermore, the study did not include molecular profiling, which is increasingly recognized as essential for accurate classification, risk stratification, and therapeutic targeting in nccRCC. The unavailability of advanced genomic testing in resource-limited settings represents a significant gap that may restrict the applicability of international guidelines. Lastly, multivariate survival analysis could not be performed due to the small number of events, limiting our ability to adjust for potential prognostic confounders. While the median follow-up period was adequate for assessing short- and medium-term outcomes, it may be insufficient to capture late recurrences and long-term survivorship, especially relevant for indolent tumors like chromophobe RCC.

Given these limitations, future research should focus on multicenter, prospective cohort studies in the Indian population to address variability in tumor biology, access to care, and long-term survival. Specific research directions may include evaluating the real-world effectiveness of emerging immunotherapy and targeted therapy regimens in nccRCC subtypes, assessing molecular and genomic profiles of Indian patients, and exploring cost-effective diagnostic and prognostic tools suitable for low-resource settings.

## Conclusions

NccRCCs represent a pathologically diverse and clinically challenging category of renal malignancies, accounting for a substantial yet underrecognized subset of kidney cancers both in India and globally. This study underscores the prognostic significance of histological subtype, with papillary and chromophobe RCCs demonstrating relatively favorable outcomes, while collecting duct carcinoma and other rare variants exhibit markedly aggressive behavior with high recurrence and mortality rates. Accurate histopathological classification, supported by immunohistochemical profiling, is critical for diagnosis, prognostication, and treatment planning. Our findings highlight the clinical utility of subtype-specific stratification and the necessity for individualized management approaches informed by tumor biology. In particular, integrating risk-adapted follow-up strategies and histology-guided adjuvant therapy may help improve outcomes in high-risk subtypes. Given the limited representation of nccRCCs in major clinical trials and the heterogeneity of these tumors, the routine incorporation of molecular profiling will be essential for refining therapeutic strategies. We advocate for the development of dedicated, evidence-based guidelines tailored to nccRCC and call for prospective, multicenter studies with larger Indian cohorts to improve generalizability. Such efforts will support the formulation of contextually relevant policy frameworks and enhance the delivery of equitable, subtype-directed oncology care in resource-limited settings like India.
